# A forgotten chapter in the history of immunotherapy: cancer therapy with *Blastomyces* extracts

**DOI:** 10.1007/s11739-024-03782-6

**Published:** 2024-11-07

**Authors:** Francesco M. Galassi, Domenico Ribatti

**Affiliations:** 1 Department of Anthropology, Faculty of Biology and Environmental Protection, University of Lodz, Łódź, Poland; 2 Department of Translational Biomedicine and Neuroscience, University of Bari Medical School, Bari, Italy

**Keywords:** Antigen, *Blastomyces*, Cancer, Immunology, Immunotherapy, Tumour

## Abstract

This article recapitulates the discoveries and anti-tumoural therapeutical proposals by Francesco Sanfelice, who in 1931 published an essay entitled *The Treatment of Cancer and Sarcoma with Cancrocidin (paraneoforming Blastomycetes)*. Sanfelice’s discoveries are contextualised with subsequent scientific discoveries, especially with those by L. Scott McDaniel and G. Cozad, who evaluated the functionality of murine peritoneal macrophages previously sensitised precisely with *Blastomyces dermatitidis* antigen extracts. Finally, recent research on the topic of intratumoural microbiota is mentioned showing how Sanfelice’s ideas, albeit partly outdated, can still inspire current biomolecular research.

Cancer represents one of the earliest known pathologies with a rich palaeopathological and fossil record [1, 2]. Fighting it has been a key priority for medicine for centuries [1, 2].

Cancer immunotherapy is based on the principle of the existence of differences between the antigenic characteristics of the tumour and those of the host within which it develops. Tumour antigens can be divided into tumour-associated foetal antigens (alpha-fetoprotein, CEA) and tumour-specific antigens, although in tumours affecting the human species no strictly specific antigenic structure has yet been highlighted. Therefore, it seems more appropriate to speak, in this case, of differentiation antigens (i.e., antigens that are present on normal cells at certain stages of their development). The host response towards the neoplastic cell population is an antibody-mediated and cell-mediated response, namely through T lymphocytes, K and NK cells, macrophages.

The difficulty of characterising tumour-specific antigens has ended up turning a nonspecific type of cancer immunotherapy that can make use of agents of synthetic or natural origin into a more feasible option. These agents act at different levels on the immune system, hence producing at the same time stimulatory and inhibitory effects: they can thus be defined as ‘immunomodulatory agents in the potentiating sense’, capable of ‘producing an end state of significant increase in the expression of immunological reactivity’ [3].

A further complicating effect in their mechanism of action derives from the very intricate network of correlations that are established within the immune system between the elements that participate in its constitution and that the said immune system establishes with other bodily systems such as the endocrine or nervous one. For this reason, it must be emphasised that the immune-potentiating effect must ‘be induced through mechanisms involving cells or products of the immune system and not only involving the nonspecific consequences of this reactivity, or those factors (e.g., endocrine, neurological, nutritional ones) that influence the function of the immune system’ [3].

In this brief note, we set out to stress the results presented in 1931 by Prof. Francesco Sanfelice, director of the Institute of Hygiene at the then Royal University of Pisa, in an Italian-language essay entitled *The Treatment of Cancer and Sarcoma with Cancrocidin (paraneoforming Blastomycetes)*. On page nine of his publication, he wrote that cancrocidin is a saline suspension of paraneoforming *Blastomycetes*, similar to pathogenic *Saccharomycetes* but lacking their pathogenic power [4]. *Blastomycetes* are fungi responsible for the onset of chronic granulomatosis that can take a circumscribed form affecting the skin and lungs, or alternatively a disseminated form [5]; a *Blastomycetes* extract, blastomycin, is currently used in delayed hypersensitivity skin tests [6]. Cancrocidin is inoculated into a vein or directly into the tumoural mass with the formation of antibodies capable of neutralising that substance (a toxin or rather an enzyme) that represents the stimulus to neoplastic proliferation. The neoplastic cells no longer being stimulated degenerate’ [4]. ‘At least sixty injections must be given, partly intravenous partly intraparenchymal, without worrying about the febrile reaction, which disappears after a few hours’ [4].

Moreover, Sanfelice wrote that ‘cure can be hoped for in recent-onset cancers. In early-onset cancers and sarcomas, in individuals in poor general condition, because of the long duration of the disease there may be improvement, but not cure’ [4]. Subsequently, Sanfelice described 53 clinical cases of patients who had experienced complete or partial regression of their tumour following treatment with cancrocidin. This is how he concluded his work: ‘All of the newly cured patients will be followed on a monthly basis and in the next work their fate will be reported, as has been done for the cured cases already described, which have been accounted for in the first part of this work’ [4].

The therapy proposed by Sanfelice in 1931, when immunology as an autonomous science had not yet been born, constituted a form of nonspecific immunotherapy of tumours that employed an agent of natural origin, just as it does today, in a phase of marked expansion of immunological research, Bacillus of Calmette and Guerin (BCG), BCG methanol extraction residue (MER), muramyl dipeptide (MDP), and *Corynebacterium parvum* are employed in cancer therapy.

From a medico-historical standpoint, Sanfelice’s theory would inevitably be shadowed by more advance approaches to cancer and its therapy in subsequent years, although it paved the way for F. Peyton Rous's discovery of oncogenic viruses that earned him the 1966 Nobel Prize in Medicine [7].

In more recent decades, specifically was in May 1983 that an article by L. Scott McDaniel and G. Cozad [8] was published in the journal *Infection and Immunity*, in which the authors reported on their study in which they evaluated the functionality of murine peritoneal macrophages previously sensitised precisely with *Blastomyces dermatitidis* antigen extracts.

The results showed an increased phagocytic capacity and metabolic activation of macrophages associated with intracellular killing of mycetes. The authors concluded their work suggesting a possible role of macrophages as modulators of the cell-mediated immune response evoked by *Blastomycetes*. In the cellular response against tumours, macrophages would naturally perform tumouricidal activity or as a result of their in vivo and in vitro exposure to chemical and biological stimuli.

Current research in this area is adding new powerful insights into how bacteria could be effectively weaponised in the fight against cancer, it having been shown that ‘engineered live therapeutics can modulate the tumour microenvironment or to deliver and selectively release therapeutic payloads to tumours’ [9]. In this perspective, it has been noted that increase of L-arginine concentrations within the tumoural formation is pivotal for anti-cancer responses of immune checkpoint inhibitors. This approach has been recently demonstrated by Canale and colleagues by adopting an engineered probiotic *Escherichia coli* Nissle 1917 strain [10], once more stressing how the metabolic pathways of the intratumoural environment can be effectively modulated. At the same time, a naïve approach could lead scientists to think the intratumoural microbiota is simply an ally of anticancer therapies, while it should be noted that it may also ‘suppress antitumour immune responses by increasing reactive oxygen species levels, creating an anti-inflammatory environment, inducing T cell inactivation, and enhancing immune suppression, thereby promoting cancer progression’ [11].

These considerations provide a further point of connection between Sanfelice’s 1931 studies and more advanced lines of research in cancer treatment, which involve the possibility of in vivo and in vitro expansion of a cytotoxic cell population aimed at facing cancer cells. 

## Data Availability

Not Applicable
